# Prevalence of Non-Volitional Sex Types and Associated Factors: A National Sample of Young People

**DOI:** 10.1371/journal.pone.0132847

**Published:** 2015-07-27

**Authors:** Nicole H. T. M. Dukers-Muijrers, Carlijn Somers, Hanneke de Graaf, Suzanne Meijer, Christian J. P. A. Hoebe

**Affiliations:** 1 Department of Sexual Health, Infectious Diseases and Environmental Health, South Limburg Public Health Service, Geleen, The Netherlands; 2 Department of Medical Microbiology, School of Public Health and Primary Care (CAPHRI), Maastricht University Medical Centre (MUMC+), Maastricht, The Netherlands; 3 Research Department, Rutgers WPF, Utrecht, The Netherlands; 4 Youth Department, STI AIDS Netherlands, Amsterdam, The Netherlands; International AIDS Vaccine Initiative, UNITED STATES

## Abstract

**Background:**

Non-volitional sex (NVS) in young people continues to be a major public health problem with long-term negative health outcomes. For the first time, the prevalence of different types of NVS and associated factors are compared between young people with same-sex sexual activities and those who have not.

**Methods:**

We obtained data from 10,401 young women and men (aged 12 to 25 years) who participated in a population study on sexual health, the Netherlands. We calculated and compared the prevalence of six types of NVS between women who had sex with men (yWSM) or women (yWSW), and men who had sex with women (yMSW) or men (yMSM). In sexually experienced participants (n = 5986) logistic regression analyses were applied to assess associations with NVS by assault or penetration. Analyses were weighted to represent the Dutch population.

**Results:**

The prevalence of NVS ranged from 1% to 61%, depending on type. Prevalence was higher for young women (any: 40.6%) than men (any: 20.4%), and highest for yMSM and yWSW. Prevalence of NVS by assault or penetration was related to a range of socio-demographic, behavioral and social factors, which were largely similar regardless of sex or same-sex-experiences. The NVS perpetrators were in over 70% of cases known to the victim; 1 in 4 cases of NVS by penetration were accompanied by violence.

**Conclusion:**

A substantial proportion of young people in the Netherlands have experienced NVS. Medical professionals, educators and caregivers should integrate services to continue to address NVS by targeting young people’s multifaceted risk profiles and evidenced based interventions for doing so are needed.

## Introduction

Non-volitional sex (NVS) is increasingly recognized as a major public health problem affecting young people and having long-term negative health outcomes [1,2]. Male and female adolescents and young adults with a history of sexual violence are at higher risk of developing a broad spectrum of psychological problems like depression [3–6], post-traumatic stress disorder or pain-related symptoms [7,8]. Further, NVS during childhood and adolescence has been associated with later high-risk sexual behavior, drinking and drug use [9–11]. Victims of NVS are also vulnerable to acquiring sexually transmitted infections (STIs) and experiencing unwanted pregnancies [12–18].

Since NVS occurs without mutual consent and against one’s will, it is a violation of fundamental human rights [19]. High prevalences of NVS are reported in the general population across the full lifespan including the young [20–22]. In 2011, 243,800 US residents age 12 or older (rate of 0.9 per 1.000) were victims of rape or sexual assault in that year [21]. A large national probability survey in the UK (NATSAL-3) reported that among sexually experienced women and men between the ages of 16 and 29 years, 16.4% experienced that someone attempted NVS and 3.7% experienced actual (completed) NVS [23]. The proportions of young people who experience NVS vary worldwide and across studies [19]. However, the direct comparison of proportions and associated factors is seriously hampered by the lack of a uniformly used measurement or definition of NVS. The UK’s Sexual Offences Act 2003 divides NVS into (1) rape, which involves penetration of the vagina, anus or mouth by a penis, (2) assault by penetration, which involves penetration of the vagina or anus by a body part (other than penis) or another object, and (3) sexual assault, which involves sexual touching of any part of the body without consent [20].

NVS is generally more often reported by young women than by young men [23–26]. Yet, men and women who had same-sex sexual experiences report the highest rates of NVS [23,27]. Next to age and same-sex activities, a wide range of factors related to socio-demographics, behavior, and social network have been associated with NVS [1–27]. Nevertheless, there is limited comparative data on the NVS experiences and associated factors in young men who have sex with women (yMSW) or men (yMSM), and women who have sex with men (yWSM) or women (yWSW).

This study uses a large Dutch population sample to examine the frequency of NVS and its associated factors among young people with and without same-sex experiences aged 12 to 25 years. Previous studies focused on forced sexual intercourse and not included other types of NVS, or included one general question about sex against one’s will. This study assesses total NVS, NVS by six different practices and NVS by two well-defined categories (i.e., by penetration and assault) [20]. We further examine their association with a wide range of factors to reveal essential new information about the prevalence of NVS by different characteristics of participants. We hope this information will signal care providers, caregivers, and educators to the occurrence of NVS, thereby targeting their attention to this multi-faceted problem in young people.

## Patients and Methods

### Procedure

Participants were recruited in two ways. Secondary school students aged 12 to 16 years came from randomly selected schools geographically spread across the Netherlands: we conducted sampling in all 12 provinces and oversampling in the southern area of the Limburg province (allowing for regional sub-analyses for local sexual health policy guidance). In addition, we recruited randomly selected individuals aged 17 to 25 years from randomly selected parts of the Municipal Personal Records Database (*Basisregistratie personen*, BRP), again with oversampling from south Limburg. Prior to the study, secondary school students received a letter at school to take home, informing parents about the study and giving them the opportunity to exclude their child from the study. Consent from parents or guardians was not documented. Participants selected from the BRP received a letter inviting them to participate.

All participants completed the questionnaire online. Secondary school participants completed the questionnaire during a regular class period, while BRP participants completed it at home or anywhere else where they had access to the internet.

The questionnaire started with written instructions including an assurance of anonymity and practical guidelines. Secondary school participants also received verbal instructions from their teachers (who had received written instructions). Authors from current paper did not interact with any of the participants nor did they have access to any participant-identifying information. The study protocol was exempt from formal medical-ethical approval under prevailing laws in the Netherlands as it concerns an observational study using anonymous questionnaire data only (as stated by the National Central Committee for Human Studies: www.ccmo.nl and in the conduct of good behavior in research www.fmwv.nl). Ethical approval was formally waived by the Medical Ethical Committee of the University Medical Center, Utrecht.

### Participants

Out of 66 randomly selected high schools (including 11 in south Limburg), 50 participated in the study (including 7 in south Limburg), a response rate of 75%. Of the selected students, 7% did not participate. Although reasons for non-participation were not recorded, according to anecdotal information from the teachers, non-participation was mainly due to sick leave of students and in some cases due to parents or students refusing participation. In addition, 46,000 young people selected from the BRP (including 13,000 from south Limburg) were invited to participate; this sample had a 16% response rate. In total, 10,401 young people in the Netherlands (including 2,560 from south Limburg) participated in this study.

### Online questionnaire and variables

#### Non-volitional sex

All participants were asked the following questions: “Have you ever experienced manual sex (‘sex by a hand’) against your will?”, “Have you ever been kissed against your will?” and “Have you ever been touched in a sexual way against your will?”. Participants who reported having had intercourse and/or anal sex were asked the following questions: “Have you ever had oral sex against your will?”, “Have you ever had sexual intercourse against your will?” and “Have you ever had anal sex against your will?”. Participants could either answer ‘yes’ or ‘no’. ‘NVS by specific practices’ was defined as an answer of ‘yes’ to any of these six questions. ‘NVS by penetration’ was defined as an answer of ‘yes’ to any or a combination of the questions about oral sex, anal sex or intercourse. ‘NVS by assault’ was defined as an answer of ‘yes’ to any or a combination of the questions about kissing, manual sex or touching only (i.e., without reporting NVS by penetration).

#### Socio-demographic, sexual health and behavioral variables

NVS is evidently associated (either as a consequence or cause) with a complex combination of individual and societal factors, several of which are assessed here. Young people who reported having had same-sex experiences (anal sex, oral sex or manual sex) were defined as yMSM (for young men) or yWSW (for young women). Other measured variables included socio-demographic factors (i.e., age, ethnicity, educational level), behavioral factors (i.e., number of sexual partners, age of sexual debut, condom use, history of STI testing, prostitution, pregnancy, perpetrating NVS), substance use before or during sex (i.e., alcohol, soft drugs, hard drugs), sexual health (i.e., satisfaction, sexual problems, knowledge, belief in ability to refuse sex), internet behavior (i.e., displaying genitals, sending own nude pictures, having cybersex, meeting sex partners, watching porn) and their social networks (i.e., number of good friends, sexual norms among friends, talking to friends about sex). Furthermore, we asked participants who reported experiencing NVS several questions about the most recent perpetrator. For the most recent perpetrator it was asked whether this was a man or a women, and whether he or she was a intimate partner, a good friend, an acquaintance (e.g. from the neighborhood), a family member, a stranger or someone else.

### Statistical analyses

We applied weighting techniques, based on current census data in the Netherlands, in data analysis to ensure a representative sample of Dutch people between the ages of 12 and 25 years in terms of sex, age, ethnic background and educational level. We calculated and compared the prevalence of total NVS, the two NVS categories, and the six specific NVS practices between young women and young men and between participants with and without same-sex experiences using chi-square statistics. Thereafter, the data were restricted to those who had had intercourse and/or anal sex (i.e. sexual experience). We assessed associations between NVS by assault and NVS by penetration and a range of factors (see variables section), stratified by young women and men using logistic regression with not having reported NVS as the reference outcome category. Weighted odds ratios (ORs) and 95% confidence intervals (CI) were calculated. All models controlled for age, educational level and ethnicity. In the model for young men, we explored interaction terms between factors and having same-sex experiences to assess whether factors differed between yMSW and yMSM. When interaction terms reached p<0.05, the main effects were reported stratified by men having same sex experiences and men who had not had such experiences. We did the same in the model for young women, exploring differences in factors between yWSM and yWSW. Further restricting data to participants who reported NVS, we described and compared the role of the perpetrator between young women and men using chi-square tests. For all main effects of the assessed associated factors, we considered p<0.01 to be statistically significant (instead of p<0.05) to compensate for the multiple comparisons. Analyses were performed using SPSS 21.0 (IBM Corporation, Somers, New York, USA).

## Results

In total, 10,401 young people filled in the questionnaire. We excluded 691 (6.6%) of them because they had missing data on any of the six NVS questions. Those who were excluded were similar to those included in terms of sex, age, region of residence and experience with anal sex. However, they were more likely (p<0.01) to have a lower educational level (60.8% versus 46.7%), non-Western ethnicity (17.2% versus 12.1%), and to have reported having intercourse (68.6% versus 61.1%). Finally, we included 3,884 young men and 5,826 young women in further analyses (S1 Table). The majority of these participants were of Western nationality and nearly half of them had a lower educational level. About two thirds of them were sexually experienced of whom about one in 10 young men or women ever had had a same sex experience.

### Prevalence of specific NVS practices

Overall, young women (40.6%) were more likely than young men (20.4%) to report having experienced any NVS (S2 Table). The prevalence of any NVS increased with age: NVS was reported by 9.0% of men aged 12–14 years, 25.6% of men aged 22–24 years, 16.6% of women aged 12–14 years and 52.7% of women aged 22–24 years. The prevalence varied between 1.2% and 60.7% by type of NVS practice, by sexual experience (S2 Table). All NVS practices were more frequently reported by young women than by young men. Young people who were sexually experienced with same-sex activities had the highest prevalence rates of non volitional touching, manual sex, oral sex, and anal sex (S2 Table). Young women who had had same-sex activities also had higher prevalences of non volitional kissing and heterosexual intercourse.

### Associated factors for NVS by assault only (without penetration)

We further assessed the factors associated with NVS by assault (without reporting any NVS by penetration) compared to not having reported NVS for sexually experienced young women and men (S3 Table and S4 Table). Risk estimates appeared to be the same for young people who reported to engage in same-sex activities and those who did not (interaction terms all p>0.05).

We observed that for both sexes, a higher prevalence of NVS by assault was associated with age between 16–20 years (compared to 12–16 years), and with a higher number of partners (S3 Table and S4 Table). In men, it was further associated with having an earlier sexual debut and previous STI testing; in women it was associated with a previous negative STI test. In both sexes, prevalence was higher when alcohol or soft drugs were used during sex The prevalence was also higher for young women who frequently had sexual problems and in those women who had at least one good friend. Further, NVS prevalence was higher in women and men who regularly talked to good friends about (how to prevent) unwanted sexual experiences.

### Associated factors for NVS by penetration

We also assessed the factors associated with NVS by penetration compared to not having reported NVS for sexually experienced young people (S3 Table and S4 Table). Risk estimates appeared to be similar between yWSM and yWSW. Three factors, indicated below, tended to be differentially associated with yMSW and yMSM (interaction terms 0.01>p<0.03).

We observed a higher prevalence of NVS by penetration in the following groups: young men with a non-Western nationality; young women and men with a lower educational level, a higher number of partners, an early sexual debut or a history of same-sex activities (S3 Table and S4 Table). Among young women, NVS by penetration was associated with a lack of condom use. Higher prevalences were observed for women and men who reported ever having been tested negative for an STI, ever receiving money or goods for sex, or ever having forced someone else into sex. Among men, prevalence was higher when they had (had) a pregnant partner. NVS was also higher in women and men who forced someone else into unwanted sex, and in women who reported the use of alcohol or soft drugs. In men, it was higher when they reported use of hard drugs. NVS by penetration was in women associated with feeling unsatisfied with their own sexual lives. In both sexes, NVS by penetration was associated with frequently having sexual problems, feeling unattractive and having sex because of fear of loosing the partner.

Feeling unable to refuse sex was associated with NVS by penetration for yMSM (26.0%, OR: 4.5, 95% CI: 1.9–10.9, p<0.01), but not for yMSW (6.6%, OR: 1.5, 95% CI: 0.9–2.4). Further, we observed a higher prevalence of NVS by penetration for young women and yMSW (17.9%, OR: 6.6, 95% CI: 4.1–10.8, p<0.01) who exposed themselves sexually in front of a webcam, but not for yMSM (20.0%, OR: 1.8, 95% CI: 0.8–4.2) who did. We also observed a higher prevalence of NVS by penetration for young women and for yMSW (23.6%, OR: 7.0, 95% CI: 4.0–12.1, p<0.01) who sent their own nude pictures over the internet, but not for yMSM (17.9%, OR: 1.7, 95% CI: 0.7–4.6) who did. The prevalence was further higher for young women and men who reported having cybersex or who had had sex with someone they met over the internet. Finally, in women, prevalence of NVS by penetration was higher when they reported to watch porn; in men it was higher when among friends it is the norm to have sex.

### Perpetrators of NVS

**Fig 1:**
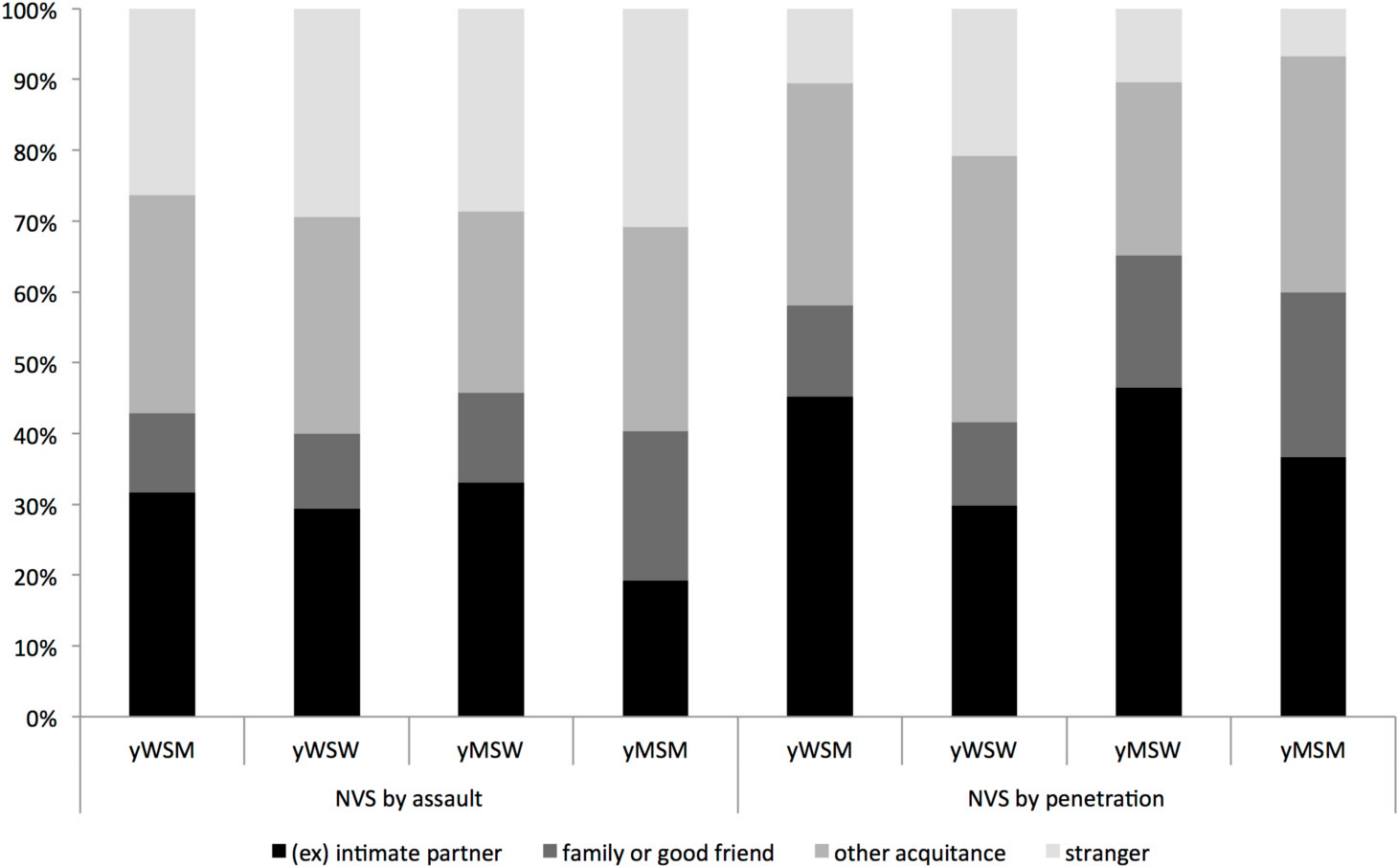
Perpetrator of NVS by assault and NVS by penetration in most recent occurrence.

Of all the sexually experienced participants who reported experiencing NVS, 93.9% filled in the question about the most recent NVS perpetrator. In most instances of NVS by penetration (88.5%) or NVS by assault (72.5%), the perpetrator was known to the victim as an intimate partner, family member, good friend, or another acquaintance; no notable differences by sex or sexual orientation were observed (Fig 1). NVS by penetration was sometimes also reported to be violent, i.e. in 32.4% for yWSM, 22.2% for yMSW, 20.0% for yMSM and 54.4% for yWSW (p<0.01 compared to all other groups).

## Discussion

This study is the first population-based sample to assess the prevalence and associated factors of different types of NVS practices and to compare them between young people who do and do not report same-sex activities. NVS is common among adolescents and young adults in the Netherlands: 1 in 2 women and 1 in 4 men who are sexually experienced and between 12 and 25 years of age reported having experienced some form of NVS. Various types of NVS were observed; the prevalences ranged from 1% to 61%. NVS by penetration was reported by 1 in 5 young women and by 1 in 20 young men. Nearly all NVS types occurred more frequently among women and men with same-sex experiences.

This study provides a comprehensive overview of NVS in young people, revealing its different practices for the first time. It shows that NVS goes beyond the strict definition of rape or coercion. While violence was involved in about 1 in 2 to 5 cases of NVS by penetration, all types of NVS represent a violation of sexual autonomy and are therefore a more subtle form of sexual violence [28]. Corroborating earlier findings, the perpetrator is usually known to the victim [5, 23, 24]. Prevalence of any NVS in our study was higher than reported in a UK population sample, which asked single questions about “attempted and completed sex against your will” [23]. This disparity is likely due to differences in the questions asked. The prevalence found in the UK study was more in line with our findings about NVS by penetration.

Corroborating earlier findings [23–27], yWSW and yMSM experienced the highest proportions of NVS, followed by yWSM. Young people with same-sex experiences were found to be more vulnerable to NVS, but also to interpersonal violence and victimization in teen dating experiences [29]. Overall, risky behaviors that are associated with adverse health outcomes were associated with experiencing NVS. For example, young people reporting NVS were more likely to report a range of risky sexual and drug use behaviors [1–18,23–26] or to engage in internet-related sexual activities [29–31]. Other associated factors were also observed: these included a lower educational level, having sexual problems, a low self-efficacy for refusing sex [2], discussing issues with friends [32], and (for young men) reporting that among their friends it was the norm to have sex. Young women and men, who had experienced NVS themselves were the most likely to have forced someone else into NVS [33,34]. The associated factors for women and men appeared to be quite similar. We also observed only few to no differences in associated factors for NVS between yMSW and yMSM, and between yWSM and yWSW. Overall, more factors were associated, and often more strongly, with NVS by penetration than with NVS by assault. Thereby, for all four groups, a multifaceted range of associated factors has been revealed that can serve as signals to target sexual health care and NVS prevention.

We recognize several limitations of this study. Due to its cross-sectional design, the study precludes drawing any causal inferences about the associations observed. Moreover, the power to detect differences between young people with and without same-sex activities may have been reduced since there were fewer yMSM and yWSW in our sample. The prevalence of NVS may have been underreported due to possible recall bias or when young people not fully disclose such information as it may be too sensitive or not fully captured in the framing of the questions [35]. There may be some age related bias in the answers to the questions due to possible age-related interpretation of the questions. Still, we do not consider it very likely that such bias would be substantial as all the questions included the terminologies used by Dutch youth and were explained in detail in the questionnaire. Although the study included a large, relatively representative sample, we do acknowledge the possibility of a partly selected sample e.g. among the young people who did obtain consent from their caregivers (versus those who did not) to participate in the study. Finally, since some of the data was collected from participants who completed the questionnaire during classes at their schools, we cannot completely rule out the possibility of group pressure to give socially desirable answers. However, most students had their own computer work spaces.

## Conclusion

This study reports on young people who have a high lifetime prevalence of NVS and for whom early intervention is essential and possible. NVS, whether by assault or penetration, was associated with a wide range of factors, corroborating previous reports and highlighting the highly clustered nature of factors associated with NVS and adverse sexual health outcomes in general [36]. NVS thus should be dealt with by interventions that address the multifaceted risk profile and that integrate existing care and prevention services.

The multifaceted risk profile, as revealed here, includes a lower educational level and a range of risky behaviours, i.e. related to sexual activity, drug use, and internet, STI test history, feeling unattractive, unsatisfied with own sexual life (women only), having sexual problems, having feelings of low self-efficacy to refuse sex (yMSM only), stating that the norm among friends to have sex (men only), and talking with friends about NVS. When these factors are encountered (either single or in combination) by educators, caregivers, and medical professionals dealing with young people, it should alert them to screen for NVS. In screening for NVS, we need to acknowledge the importance of asking about the different types of NVS experiences. Yet, in practice it may not be easy to address NVS either directly or indirectly, by its associated factors, due to the sensitive nature of these topics. Youth who have NVS experiences may not typically disclose them [23]. There is a growing understanding of NVS intervention strategies that may be useful in various settings (e.g., in communities and schools [37], using the internet or in clinical settings [36]). However, much remains to be understood about the specific social context that links NVS to the interventions and about the interventions that are most effective [36,37]. For example, the mainly heteronormative clinical and school environments may exacerbate health inequities for young people who engage in same-sex sexual activities [36,38]. To address NVS effectively and with integrated services, we urgently need carefully designed evidenced based interventions. The associated factors revealed in our current study can be an important starting point for systematic development of such interventions (e.g. by using intervention mapping [39]) including their successful implementation in educational, prevention and clinical contexts.

## Supporting Information

S1 Table Basic characteristics of the study sample. (DOCX)

S2 Table Weighted prevalences of NVS practices in 12–25 year old women and men by sexual experience and same-sex activities, Netherlands. (DOCX)

S3 Table Weighted prevalence of non-volitional sex (NVS) by assault and NVS by penetration and associations by demographic, health and behavioural factors compared to volitional sex in sexually experienced young women. (DOCX)

S4 Table Weighted prevalence of non-volitional sex (NVS) by assault and NVS by penetration and associations by demographic, health and behavioural factors compared to volitional sex in sexually experienced young men. (DOCX)
