# Supplementary material for: Prevalence of Non-Volitional Sex Types and Associated Factors: A National Sample of Young People
Source: PLoS One. 2015 Jul 27;10(7):e0132847. doi: 10.1371/journal.pone.0132847 (PMC4516263; doi:10.1371/journal.pone.0132847)
Supplement: S1 Table — (DOCX) [file pone.0132847.s001.docx]

S1 Table. Basic characteristics of the study sample

|  | **Young women (N=5826)** | **Young men (N=3884)** |
| --- | --- | --- |
|  | **% (n)** | **% (n)** |
| Mean age (years) | 18.6 | 18.4 |
| Dutch or other Western ethnicity | 88.3 (5142) | 87.3 (3389) |
| Low educational level | 47.3 (2758) | 45.8 (1780) |
| Mean age sexual debut (years) | 16.1 | 16.4 |
| Any same-sex activities (ever) | 7.5 (438) | 6.4 (248) |
| Sexually experienced (intercourse and/or any anal sex ever) | 63.3 (3686) | 59.2 (2300) |
| Any same-sex activities among sexually experienced young people | 10.4 (384) | 9.8 (226) |
