# Supplementary material for: Prevalence of Non-Volitional Sex Types and Associated Factors: A National Sample of Young People
Source: PLoS One. 2015 Jul 27;10(7):e0132847. doi: 10.1371/journal.pone.0132847 (PMC4516263; doi:10.1371/journal.pone.0132847)
Supplement: S2 Table — (DOCX) [file pone.0132847.s002.docx]

S2 Table. Weighted prevalences of NVS practices in 12-25 year old women and men by sexual experience and same-sex activities, Netherlands

| Type of NVS | All young women | | yWSM^ | | yWSW^ |  | All young men | yMSW^ | yMSM^ |
| --- | --- | --- | --- | --- | --- | --- | --- | --- | --- |
|  | *SEXUALLY EXPERIENCED* | | | | | | | | |
|  | unweighted | | (unweighted | (unweighted | |  | (unweighted | (unweighted | (unweighted |
|  | N=3686; weighted | | N=3302; weighted | N=384; weighted | |  | N=2300; weighted | N=2074; weighted | N=226; weighted |
|  | N=2227) | | N=1988) | N=239) | |  | N=2171) | N=1951) | N=220) |
| Kissing | 30.0% | | 28.3% | 44.8%** | |  | 24.4%* | 20.1% | 22.7% |
| Touching | 43.3% | | 41.2% | 60.7%** | |  | 14.8%* | 13.9% | 22.7%** |
| Manual sex | 15.8% | | 15.0% | 22.2%** | |  | 4.5%* | 3.9% | 10.0%** |
| Oral sex | 12.2% | | 11.4% | 19.7%** | |  | 3.5%* | 3.0% | 8.6%** |
| Heterosexual intercourse | 13.9% | | 12.4% | 25.9%** | |  | 3.2%* | 3.2% | 3.6% |
| Anal sex | 5.1% | | 4.4% | 10.9%** | |  | 3.3%* | 2.6% | 10.0%** |
| *Any NVS by assault@* | 35.6% | | 35.5% | 36.0% | |  | 22.9%* | 22.8% | 24.1% |
| *Any NVS by penetration#* | 20.3% | | 18.9% | 32.2%** | |  | 5.7%* | 4.8% | 13.6%** |
| *Any NVS* | 55.9% | | 54.4% | 68.2%** | |  | 28.6%* | 27.5% | 37.7%** |
|  | *SEXUALLY IN-EXPERIENCED* | | | | | | | | |
|  | unweighted | (unweighted | | | (unweighted |  | (unweighted | (unweighted | (unweighted |
|  | N=2140; weighted | N=2086; weighted | | | N=54; weighted |  | N=1584; weighted | N=1562; weighted | N=22; weighted |
|  | N=1877) | N=1837) | | | N=41) |  | N=1801) | N=1779) | N=21) |
| Kissing | 11.1% | 10.7% | | | 25.0% |  | 6.6%* | 6.4% | 23.8% |
| Touching | 16.5% | 16.1% | | | 34.1%** |  | 6.2%* | 6.1% | 19.0% |
| Manual sex | 4.5% | 4.2% | | | 17.1%** |  | 1.3%* | 1.2% | 9.1% |
| *Any NVS by assault@* | 22.5% | 22.1% | | | 41.5%** |  | 10.5%* | 10.3% | 27.3% |
|  | *ALL (SEXUALLY EXPERIENCED AND IN-EXPERIENCED)* | | | | | | | | |
|  | unweighted | (unweighted | | (unweighted | |  | (unweighted | (unweighted | (unweighted |
|  | N=5826; weighted | N=5388; weighted | | N=438; weighted | |  | N=3884; weighted | N=3636; weighted | N=248; weighted |
|  | N=4104) | N=3825) | | N=279) | |  | N=3972) | N=3730) | N=242) |
| *Any NVS by assault@* | 29.6% | 29.1% | | 36.8% | |  | 17.3%* | 16.8% | 24.4% |
| *Any NVS by penetration#* | 11.0% | 9.8% | | 27.6% | |  | 3.1%* | 2.5% | 12.4% |
| *Any NVS* | 40.6% | 38.9% | | 64.5% | |  | 20.4%* | 19.3% | 36.8% |

^ young people who never reported any same-sex activities yWSM young women who have sex with men and yMSW young men who have sex with women; and young people who ever reported any same-sex activities yWSW young women who have sex with women yMSM young men who have sex with men

~Sexually experienced: reported practice of heterosexual sexual intercourse and/or anal sex

@ NVS by assault included any or a combination of NVS by kissing, touching, or manual sex without report of NVS by oral sex, anal sex or intercourse

# NVS by penetration included any or a combination of NVS by oral sex, anal sex or intercourse

* P<0.01 in Chisquare test on difference between young men and women

** P<0.01 in Chisquare test on difference between young people with and without same-sex-activities for young men and women separately
