# Supplementary material for: Prevalence of Non-Volitional Sex Types and Associated Factors: A National Sample of Young People
Source: PLoS One. 2015 Jul 27;10(7):e0132847. doi: 10.1371/journal.pone.0132847 (PMC4516263; doi:10.1371/journal.pone.0132847)
Supplement: S3 Table — (DOCX) [file pone.0132847.s003.docx]

S3 Table. Weighted prevalence of non-volitional sex (NVS) by assault and NVS by penetration and associations by demographic, health and behavioural factors compared to volitional sex in sexually experienced young women

|  | **NVS by assault** | | **NVS by penetration** | |
| --- | --- | --- | --- | --- |
|  | **%** | **OR(95% CI)** | **%** | **OR(95% CI)** |
| **Demographics** |  |  |  |  |
| Age |  |  |  |  |
| 12-15 | 24.6 | 1 | 17.8 | 1 |
| 16-20 | 38.0 | 2.1 (1.3-3.3)* | 20.2 | 1.6 (1.0-2.7) |
| 21-24 | 34.3 | 1.8 (1.1-3.0) | 20.7 | 1.6 (1.0-2.7) |
| Ethnicity |  |  |  |  |
| Dutch or other Western | 36.2 | 1 | 19.3 | 1 |
| Non-western | 31.5 | 0.9 (0.7-1.3) | 27.0 | 1.4 (1.1-2.0) |
| Educational level |  |  |  |  |
| Middle/high | 37.7 | 1 | 15.4 | 1 |
| Low | 34.0 | 1.0 (0.8-1.2) | 24.0 | 1.7 (1.4-2.2)* |
| **Sexual behaviour** |  |  |  |  |
| Lifetime number of sex partners |  |  |  |  |
| 1 | 34.2 | 1 | 9.2 | 1 |
| 2-3 | 39.1 | 1.5 (1.2-1.9)* | 19.5 | 3.0 (2.1-4.1)* |
| 4 or more | 33.9 | 1.7 (1.4-2.2)* | 32.5 | 6.3 (4.6-8.6)* |
| Sexual debut |  |  |  |  |
| 16 years old or older | 36.2 | 1 | 16.4 | 1 |
| 15 years old or younger | 34.6 | 1.3 (1.0-1.3) | 26.5 | 2.2 (1.7-2.8)* |
| Same-sex activities (ever) |  |  |  |  |
| No (yWSM) | 35.5 | 1 | 18.9 | 1 |
| Yes (yWSW) | 36.0 | 1.1 (1.0-1.3) | 32.2 | 1.4 (1.2-1.5)* |
| Use of condom with most recent partner |  |  |  |  |
| No | 35.6 | 1.1 (0.9-1.4) | 21.5 | 1.6 (1.1-1.2)* |
| Yes | 35.7 | 1 | 15.5 | 1 |
| History of STI testing |  |  |  |  |
| Tested positive | 29.6 | 1.2 (0.6-2.3) | 37.0 | 2.2 (1.1-4.3) |
| Tested negative | 41.3 | 1.6 (1.3-2.1)* | 25.3 | 1.7 (1.3-2.3)* |
| No STI test | 34.5 | 1 | 18.7 | 1 |
| Ever received money or goods for sex |  |  |  |  |
| No | 35.6 | 1 | 19.3 | 1 |
| Yes | 18.8 | 1.1 (0.5-2.5) | 59.4 | 6.2 (3.3-11.7)* |
| Ever had partner who was pregnant |  |  |  |  |
| No | 36.0 | 1 | 19.8 | 1 |
| Yes | 26.1 | 0.8 (0.5-1.2) | 31.3 | 1.3 (0.8-2.1) |
| Ever forced someone else into sex |  |  |  |  |
| No | 35.6 | 1 | 20.0 | 1 |
| Yes | 31.3 | 3.0 (0.6-15.7) | 56.3 | 9.6 (2.1-44.5)* |
| **Substance use before/during sex (ever)** |  |  |  |  |
| Alcohol |  |  |  |  |
| No | 31.9 | 1 | 16.7 | 1 |
| Yes | 37.1 | 1.4 (1.1-1.7)* | 21.9 | 1.8 (1.4-2.3)* |
| Soft-drugs |  |  |  |  |
| No | 34.9 | 1 | 18.0 | 1 |
| Yes | 39.2 | 1.9 (1.5-2.6)* | 33.5 | 3.1 (2.2-4.1)* |
| Hard-drugs |  |  |  |  |
| No | 35.8 | 1 | 19.9 | 1 |
| Yes | 27.5 | 1.1 (0.5-2.2) | 39.2 | 2.4 (1.2-4.6) |
| **Sexual health** |  |  |  |  |
| Unhappy with own sexual life |  |  |  |  |
| Disagree/neutral | 35.9 | 1 | 19.3 | 1 |
| Agree | 31.5 | 1.1 (0.8-1.7) | 33.5 | 2.4 (1.6-3.5)* |
| Regularly has sexual problems |  |  |  |  |
| No | 33.0 | 1 | 17.4 | 1 |
| Yes | 39.4 | 1.6 (1.3-2.0)* | 24.6 | 2.0 (1.6-2.5)* |
| Knowledge score on 7 sexual health items |  |  |  |  |
| 4 or less correct | 33.3 | 0.9 (0.7-1.1) | 21.5 | 1.0 (0.8-1.3) |
| 5 or more correct | 36.4 | 1 | 19.9 | 1 |
| Felt unattractive |  |  |  |  |
| Disagree/neutral | 36.2 | 1 | 18.6 | 1 |
| Agree | 31.7 | 1.1 (0.8-1.4) | 30.2 | 2.0 (1.5-2.7)* |
| Felt unable to refuse sex when someone is persuasive |  |  |  |  |
| Disagree/neutral | 36.1 | 1 | 19.4 | 1 |
| Agree | 31.4 | 1.0 (0.7-1.4) | 28.5 | 1.6 (1.1-2.2) |
| Had sex because of fear of loosing the partner |  |  |  |  |
| Disagree/neutral | 36.2 | 1 | 19.1 | 1 |
| Agree | 30.1 | 1.0 (0.7-1.4) | 30.5 | 1.8 (1.3-2.5)* |
| **Internet behavior** |  |  |  |  |
| Ever showed genitals in front of webcam |  |  |  |  |
| No | 35.7 | 1 | 19.5 | 1 |
| Yes | 33.6 | 1.2 (0.8-1.8) | 32.9 | 2.0 (1.3-3.1)* |
| Ever send own nude picture/films |  |  |  |  |
| No | 35.6 | 1 | 19.4 | 1 |
| Yes | 35.2 | 1.6 (1.0-2.4) | 35.2 | 2.7 (1.7-4.3)* |
| Ever had sex on the internet (cybersex) |  |  |  |  |
| No | 35.7 | 1 | 19.5 | 1 |
| Yes | 31.6 | 1.4 (0.8-2.4) | 38.9 | 3.1 (1.9-5.2)* |
| Ever had sex with someone met by the internet |  |  |  |  |
| No | 35.9 | 1 | 18.9 | 1 |
| Yes | 29.5 | 1.4 (0.9-2.2) | 43.2 | 3.5 (2.3-5.4)* |
| Watched porn on the internet (past 6 months) |  |  |  |  |
| No | 34.3 | 1 | 18.3 | 1 |
| Yes | 38.5 | 1.1 (0.5-2.5) | 25.2 | 6.2 (3.3-11.7)* |
| **Social network** |  |  |  |  |
| Current number of good friends |  |  |  |  |
| 0 | 23.3 | 1 | 24.7 | 1 |
| 1 | 37.7 | 2.3 (1.2-4.5)* | 25.0 | 1.5 ((0.8-3.0) |
| 2 | 39.3 | 2.3 (1.2-4.2)* | 21.5 | 1.3 (0.7-2.5) |
| 3 or more | 34.4 | 1.7 (0.9-3.1) | 19.0 | 1.1 (0.6-2.0) |
| The norm of my friends is to have sex |  |  |  |  |
| Disagree/neutral | 36.2 | 1 | 20.0 | 1 |
| Agree | 28.8 | 0.8 (0.5-1.5) | 24.4 | 1.1 (0.6-2.1) |
| I talk to my friends about sexual things I do not want to do |  |  |  |  |
| Never/sometimes | 33.4 | 1 | 20.6 | 1 |
| Regularly | 38.5 | 1.3 (1.1-1.6)* | 20.0 | 1.0 (0.8-1.3) |
| I talk to my friends how to prevent negative sexual experiences |  |  |  |  |
| Never/sometimes | 35.1 | 1 | 19.7 | 1 |
| Regularly | 36.7 | 1.1 (0.9-1.4) | 21.7 | 1.1 (0.9-1.5) |

OR: Odds Ratio adjusted for age, educational level, ethnicity; CI: Confidence Interval

* p<0.01
