# Supplementary material for: Prevalence of Non-Volitional Sex Types and Associated Factors: A National Sample of Young People
Source: PLoS One. 2015 Jul 27;10(7):e0132847. doi: 10.1371/journal.pone.0132847 (PMC4516263; doi:10.1371/journal.pone.0132847)
Supplement: S4 Table — (DOCX) [file pone.0132847.s004.docx]

S4 Table. Weighted prevalence of non-volitional sex (NVS) by assault and NVS by penetration and associations by demographic, health and behavioural factors compared to volitional sex in sexually experienced young men

|  | **NVS by assault** | | **NVS by penetration** | |
| --- | --- | --- | --- | --- |
|  | **% (n)** | **OR(95% CI)** | **% (n)** | **OR(95% CI)** |
| **Demographics** |  |  |  |  |
| Age |  |  |  |  |
| 12-15 | 15.1 | 1 | 9.7 | 1 |
| 16-20 | 24.8 | 1.8 (1.2-2.7)* | 5.9 | 0.6 (0.3-1.0) |
| 21-24 | 22.4 | 1.6 (1.0-2.4) | 4.6 | 0.7 (0.4-1.2) |
| Ethnicity |  |  |  |  |
| Dutch or other Western | 23.8 | 1 | 4.2 | 1 |
| Non-western | 17.7 | 0.8 (0.6-1.1) | 14.1 | 3.3 (2.2-4.8)* |
| Educational level |  |  |  |  |
| Middle/high | 21.7 | 1 | 2.9 | 1 |
| Low | 23.6 | 1.2 (0.9-1.5) | 7.3 | 2.4 (1.5-3.8)* |
| **Sexual behaviour** |  |  |  |  |
| Lifetime number of sex partners |  |  |  |  |
| 1 | 18.5 | 1 | 1.9 | 1 |
| 2-3 | 21.8 | 1.3 (1.0-1.7) | 6.2 | 3.6 (1.9-6.9)* |
| 4 or more | 27.7 | 2.0 (1.5-2.5)* | 8.6 | 5.4 (2.8-10.1)* |
| Sexual debut |  |  |  |  |
| 16 years old or older | 22.0 | 1 | 4.0 | 1 |
| 15 years old or younger | 24.4 | 1.4 (1.1-1.7)* | 8.5 | 1.9 (1.2-2.8)* |
| Same-sex activities (ever) |  |  |  |  |
| No (yMSW) | 22.8 | 1 | 4.8 | 1 |
| Yes (yMSM) | 24.1 | 1.3 (0.9-1.8) | 13.6 | 3.4 (2.2-5.4)* |
| Use of condom with most recent partner |  |  |  |  |
| No | 36.6 | 1.1 (0.9-1.4) | 5.4 | 1.3 (0.8-1.9) |
| Yes | 21.6 | 1 | 6.2 | 1 |
| History of STI testing |  |  |  |  |
| Tested positive | 34.4 | 2.8 (1.3-6.4) | 25.0 | 6.2 (2.4-15.9)* |
| Tested negative | 25.4 | 1.1 (0.9-1.5) | 6.7 | 3.2 (2.0-5.1)* |
| No STI test | 22.5 | 1 | 5.2 | 1 |
| Ever received money or goods for sex |  |  |  |  |
| No | 21.6 | 1 | 4.1 | 1 |
| Yes | 35.1 | 1.2 (0.9-1.4) | 24.3 | 2.9 (1.9-4.4)* |
| Ever had partner who was pregnant |  |  |  |  |
| No | 22.5 | 1 | 4.6 | 1 |
| Yes | 25.4 | 1.6 (1.0-2.5) | 18.4 | 4.6 (2.6-8.0)* |
| Ever forced someone else into sex |  |  |  |  |
| No | 22.7 | 1 | 4.8 | 1 |
| Yes | 26.9 | 1.7 (1.1-2.8) | 22.2 | 6.8 (3.9-11.7)* |
| **Substance use before/during sex (ever)** |  |  |  |  |
| Alcohol |  |  |  |  |
| No | 16.0 | 1 | 7.5 | 1 |
| Yes | 25.6 | 1.7 (1.3-2.2)* | 4.9 | 1.0 (0.7-1.6) |
| Soft-drugs |  |  |  |  |
| No | 20.4 | 1 | 5.3 | 1 |
| Yes | 31.5 | 1.9 (1.5-2.4)* | 7.1 | 1.5 (1.0-2.3) |
| Hard-drugs |  |  |  |  |
| No | 23.0 | 1 | 5.2 | 1 |
| Yes | 21.0 | 1.2 (0.7-2.1) | 18.5 | 3.2 (1.7-6.1)* |
| **Sexual health** |  |  |  |  |
| Unhappy with own sexual life |  |  |  |  |
| Disagree/neutral | 23.0 | 1 | 5.2 | 1 |
| Agree | 22.0 | 1.0 (0.7-1.4) | 9.9 | 1.9 (1.2-2.3) |
| Regularly has sexual problems |  |  |  |  |
| No | 22.5 | 1 | 4.7 | 1 |
| Yes | 25.8 | 1.4 (1.0-1.9) | 14.2 | 3.6 (2.3-5.6)* |
| Knowledge score on 7 sexual health items |  |  |  |  |
| 4 or less correct | 22.7 | 1.1 (0.9-1.3) | 8.0 | 1.6 (1.1-2.3) |
| 5 or more correct | 23.0 | 1 | 4.0 | 1 |
| Felt unattractive |  |  |  |  |
| Disagree/neutral | 23.3 | 1 | 4.8 | 1 |
| Agree | 18.9 | 0.9 (0.6-1.3) | 14.8 | 3.6 (2.2-5.7)* |
| Felt unable to refuse sex when someone is persuasive |  |  |  |  |
| Disagree/neutral | 22.4 | 1 | 4.8 | 1 |
| Agree | 24.6 | 1.2 (1.0-1.5) | 8.6 | 1.8 (1.2-2.7)*@ |
| Had sex because of fear of loosing the partner |  |  |  |  |
| Disagree/neutral | 21.9 | 1 | 5.0 | 1 |
| Agree | 27.0 | 1.4 (1.1-1.8) | 8.5 | 1.8 (1.2-2.7)* |
| **Internet behavior** |  |  |  |  |
| Ever showed genitals in front of webcam |  |  |  |  |
| No | 22.7 | 1 | 4.8 | 1 |
| Yes | 24.6 | 1.4 (1.0-1.9) | 18.1 | 5.7 (3.7-8.6)*@ |
| Ever send own nude picture/films |  |  |  |  |
| No | 23.1 | 1 | 4.5 | 1 |
| Yes | 20.5 | 1.2 (0.8-1.8) | 21.9 | 5.9 (3.7-9.4)*@ |
| Ever had sex on the internet (cybersex) |  |  |  |  |
| No | 22.7 | 1 | 4.5 | 1 |
| Yes | 24.1 | 1.3 (0.9-1.8) | 15.1 | 4.0 (2.6-6.2)* |
| Ever had sex with someone met by the internet |  |  |  |  |
| No | 23.1 | 1 | 4.2 | 1 |
| Yes | 21.9 | 1.1 (0.8-1.5) | 15.2 | 3.2 (2.1-4.9)* |
| Watched porn on the internet (past 6 months) |  |  |  |  |
| No | 19.6 | 1 | 5.2 | 1 |
| Yes | 23.7 | 1.3 (1.0-1.7) | 5.8 | 1.5 (0.9-2.4) |
| **Social network** |  |  |  |  |
| Current number of good friends |  |  |  |  |
| 0 | 11.5 | 1 | 7.7 | 1 |
| 1 | 14.7 | 1.3 (0.5-3.6) | 9.1 | 1.7 (0.5-5.7) |
| 2 | 23.4 | 2.3 (0.9-5.6) | 5.4 | 1.1 (0.3-3.6) |
| 3 or more | 23.9 | 2.4 (1.0-5.9) | 5.4 | 1.2 (0.4-3.6) |
| The norm of my friends is to have sex |  |  |  |  |
| Disagree/neutral | 23.6 | 1 | 4.8 | 1 |
| Agree | 19.1 | 1.0 (0.6-1.5) | 13.7 | 2.5 (1.4-4.4)* |
| I talk to my friends about sexual things I do not want to do |  |  |  |  |
| Never/sometimes | 20.6 | 1 | 5.0 | 1 |
| Regularly | 27.3 | 1.5 (1.2-1.9)* | 7.0 | 1.4 (1.0-2.1) |
| I talk to my friends how to prevent negative sexual experiences |  |  |  |  |
| Never/sometimes | 21.9 | 1 | 4.9 | 1 |
| Regularly | 26.4 | 1.4 (1.1-1.8)* | 8.2 | 1.5 (1.0-2.3) |

OR: Odds Ratio adjusted for age, educational level, ethnicity; CI: Confidence Interval

@ risk estimates were different for yMSW and yMSM (p interaction-term<0.03)

* p<0.01
